# Supplementary material for: Activity in perceptual classification networks as a basis for human subjective time perception
Source: Nat Commun. 2019 Jan 17;10:267. doi: 10.1038/s41467-018-08194-7 (PMC6336826; doi:10.1038/s41467-018-08194-7)
Supplement: Supplementary file 1 — Supplementary Information [file 41467_2018_8194_MOESM1_ESM.pdf]

Supplementary Information

Activity in perceptual classification networks as a basis for human subjective time perception

Roseboom *et al.*

1 Supplementary Discussion

Content, not model regularity drives time estimation A potential criticism of the results in the main text would be that they simply reflect the operation of another type of pacemaker, in this case one that underlies the updating of perceptual content. As calculation of salient network activation changes in the model occurs at some defined frequency (the video was input to the system, and activation difference calculated, at 30 Hz in the above results), one might suspect that our system is simply mimicking a physical pacemaker, with the regular updates taking the role of, in the most trivial example, the movement of hands on a clock face. However, it is easy to demonstrate that the regularity of model operation is not the predominant feature in determining time estimates. If it were, duration estimates for the "Gaze" versus "Shuffled" models would be highly similar, as they contain the same input rate (30 Hz) and temporal features induced by movement of the gaze spotlight. This is clearly not the case (Fig. 3c and Fig. 3d in main text).

To thoroughly reject the concern that the regularity of the system update rate was the main determinant of time estimation in our system, we compared the salient changes accumulated by the system when inputting the "normal" videos at 30 Hz, with accumulated changes under three conditions: videos in which the frame rate was halved (skipped every second frame), videos in which some frames were skipped pseudo-randomly with a frequency of 20%, or videos input at 30Hz, but with the video frames presented in a random order. The results showed that the manipulations of frame rate (skipping every second frame or 20% of frames) produced only small differences in accumulated changes over time compared to the normal input videos Supplementary (Fig. 2). However, when the input rate was kept at 30 Hz, but with a random presentation order of frames, thereby disrupting the flow of content in the video, the number of accumulated changes was very different (up to around 40 times *more* different from standard than either the halved or randomly skipped frame cases; see Supplementary Fig. 2).

If the system update rate was simply acting as a pacemaker we would expect that the accumulation of salient perceptual changes would change proportionally to the change in update rate. This was clearly not the case at the basic level of the change accumulation (even before mapping these accumulations into duration labels using support vector regression). Only when the content of the scene was changed, by altering the order in which frames were presented but keeping the standard 30 Hz update rate, was there a large change in accumulated salient changes. Therefore, these results underline that our system was producing temporal estimates based on the content of the scene, not the update rate of the system.

Model performance is robust across threshold parameters The parameters of the model, T^k_{max}, T^k_{min} and τ^k, were chosen so that the Euclidean distances for each layer exceeded the threshold only when a large increase occurred. The choice of particular values is not very important as the model performance is robust across a broad range of these values. When we scaled the values of T^k_{max} and T^k_{min} by a factor allowing us to vary the level of the threshold mechanism (*attention modulation*), our model could still estimate time with relatively good accuracy across a broad range of parameter values (Supplementary Fig. 3a-c) and, most importantly, still differentiate between short and long durations (slope is greater than zero for most levels). To further examine the effect of T^k_{max} and T^k_{min}, we scaled each parameter by an independent scaling factor to show that the model estimations (compared to the real physical duration) are robust over a wide range of values for these two parameters (Supplementary Fig. 3d-f). These results show that system-produced estimation is relatively accurate (relative to physical duration) across a very broad range of parameters for T^k_{max} and T^k_{min}.

Model performance does not depend on threshold decay The threshold used in the experiments reported in the main text included a noisy decay that continued until the threshold was exceeded, according to the parameters described in Equation 1. This decay was included to approximate the role of normalisation of neural response that is known to occur within the sensory systems (e.g. visual processing) the function of which we are attempting to mimic, and further, to facilitate model performance across a wider array of possible scene types and content. However, this decay is not essential for the model to perform well, discriminate short from long durations, and have the potential for attentional modulation. This can be seen if the threshold is set at a single level for all scenes and the regression mechanism is trained on accumulated salient changes under this single threshold level. As shown in Fig. 4, if the threshold is simply fixed as

$$T^k_{\text{t+1}} = T^k_{\text{fixed}} = \frac{T^k_{\text{max}} + T^k_{\text{min}}}{2} \tag{1}$$

then the estimation remains similar to that reported in the main text (e.g. Fig. 3b-c). Furthermore, as discussed in the section **Accounting for the role of attention in time perception** in the main text, if this threshold level is changed by modulation of a global scaling factor $C > 0$ of *attention modulation*, system duration estimates become biased. In this case, the impact on the threshold when modulating attention can be seen as $T^k_{\text{t+1}} \leftarrow C \cdot T^k_{fixed}$, thus altering the probability that a given change between consecutive frames will be determined to be salient and accumulated to drive an increase in subjective duration. As a result, estimations become biased towards shorter estimations with a lower attention level, and longer estimations with higher attention level - consistent with the proposed interaction of attention and duration estimation covered in the main text.

This effect shows that the dynamic nature of the threshold in the main implementation is not strictly necessary for meaningful estimates of time to be generated when tracking salient changes in network activation, and for those estimates to be modulated by attention to time.

Model performance is not due to regression overfitting As specified in the Methods, the number of accumulated salient perceptual changes recorded in the accumulators represent the elapsed duration between two points in time. In order to convert estimates of subjective time into units of time in seconds, a simple regression method was used based on epsilon-Support Vector Regression (SVR) from scikit-learn python toolkit[1]. The kernel used was the radial basis function with a kernel coefficient of 10^{-4} and a penalty parameter for the error term of 10^{-3}. We used 10-fold cross-validation. To produce the presented data, we used 9 out of 10 groups for training and one (i.e. 10% of data) for testing. This process was repeated 10 times so that each group was used for validation only once. In order to verify that our system performance was not simply due to overfitting of the regression method for the set of included durations, rather than the ability of the system to estimate time, we tested the model estimation performance when excluding some durations from the training set, but keeping them in the testing set. The mean normalised error for durations included and excluded in each experiment is shown in (Supplementary Fig. 5). As can be seen, only when excluding a large number of training levels (e.g. 10 out of 13 possible levels) does the estimation error get notably larger, suggesting that model performance is not attributable only to overfitting in the regression - duration estimates are robust across the tested range.

Changes in classification network activation, not just stimulation, are critical to human-like time estimation As outlined in the Introduction, our proposal is built on the idea that changes in the sensory environment, as reflected by neural activation within sensory processing networks, provide a mechanistic basis for human time perception. In a minimal interpretation, one might suspect that the efficacy of our model (including the basic ability of the model to estimate time, that model-produced estimates improve with human-like gaze constraints, and that estimates are biased in different scenes in a way that follows human reports) may reflect only the basic stimulus properties. This interpretation would mean that the use of a human-like sensory classification network adds little to our understanding of duration estimation generally, or more precisely, the role of sensory classification networks in human time perception. To examine this issue we conducted a series of experiments wherein, rather than using the difference in network activation to indicate salient difference, we directly measured the Euclidean distance, by pixel, between successive frames of the stimulus videos. As in the initial experiments reported in the main text, we conducted these experiments under two conditions: one condition in which each frame of the video was constrained by human gaze data ("Gaze"), and another condition in which the whole video frame was used (Full-frame). In both cases, as with the initial experiments, the difference between successive frames was compared to a dynamic threshold, detected salient differences accumulated during a test epoch, and support vector regression trained on the accumulated salient differences and the physical labels of the interval in order to produce estimates of duration in seconds (as detailed in the methods for the main model). Consequently, any potential difference in results between these experiments and the experiments reported in the main text, conducted based on activations within the classification network, indicate the contribution of the perceptual processing within the classification network to time perception.

As can be seen in Supplementary Fig. 7, estimates of duration can still be produced based on the pixel-wise differences in the video for both "Gaze" constrained video, as well as for the Full-frame video, as indicated by non-zero slopes in estimation. This basic sensitivity to duration is not surprising, given that our model of time perception is based on perceptual changes driven by sensory signals. Crucially, though, these results show several clear differences to both the classification network-based estimates, as well as human reports. Most obviously, estimation when using the Full-frame video is much poorer than for either of the "Gaze" or Full-frame models reported in the main text, with short durations dramatically overestimated, and estimations for office and cafe scenes similarly underestimated. These findings are clearly reflected in the mean deviation of estimation, shown in Supplementary Fig. 6. While the overall pattern of biases by scene for the "Full-frame" video replicate the same pattern as for human reports (city > campus/outside > cafe/office; see Fig. 3g in the main text), both the overestimation of scenes containing more change (city and campus/outside) and the underestimation of the scenes containing less change (office/cafe) are much more severe. Overall, poor estimation performance when using "Full-frame" video is attributable to the estimation being driven only by the pixel-wise changes in the scene, especially for scenes wherein very little changes between successive frames on a pixel-wise basis (office/cafe; green line in Supplementary Fig. 7). In these scenes, there are many instances where the scene remains unchanged for extended periods, therefore producing no pixel-wise differences at all with which to drive estimation.

By contrast, estimations based on gaze-constrained video ("Gaze" input) show superior performance to those for the Full-frame input video, with better slope of estimation (Supplementary Fig. 7), and less severe over/underestimation. These results support the findings reported in the main text regarding the importance of where humans look in a scene to the estimation of duration. However, as is clearly depicted in Supplementary Fig. 6, when considering the pattern of biases induced by different scenes, estimations based only on gaze-constrained video do not replicate the pattern of results seen for both the

classification network-based model and human estimations (Fig. 3g and h) reported in the main text. Rather, estimations based on gaze-constrained video alone substantially underestimate durations for scenes based in the campus/outside, while overestimating the scenes with the least perceptual change (office/cafe scenes).

Overall, these results show, consistent with the proposal outlined in the Introduction, that the basis for human-like time perception can be simply located within changes in sensory stimuli. More importantly, they also show that it is not just the sensory stimuli alone that drive time perception, but also how stimulation is interpreted within perceptual classification networks. By basing our core model, as reported in the main text, on stimulus-driven activation in a human-like visual classification network, our model is able to naturally capture human-like biases in duration estimation in a way that is not possible based on the sensory stimuli alone.

Estimate variance by duration That the variability of duration estimates is proportional to the presented duration is often cited as a key feature of time perception and referred to as scalar variability (e.g.[2]). The more general case is described by Weber's law. In both cases, the key feature in the data is that estimation is proportionally more variable for larger values of the stimulus intensity (duration in this case). In the main text Results section **Tracking changes in perceptual classification produces human-like time estimation** and (Fig. 3a-d in the main text) it is reported that both our human observers and our model produced estimates appear to follow scalar variability. As the duration estimates depicted in Fig. 3a-d in the main text are shown on a log-log scale and the shaded area around the mean line indicates ± 1 standard deviation of the mean, the roughly constant size of this shaded area across levels of duration can be taken as evidence for scalar variability in these estimates. To provide further evidence in favour of this interpretation, we calculated the coefficient of variation (CoV; the standard deviation of estimation divided by the mean estimation) for each level of duration estimated by our human participants and for the three model configurations presented in Fig. 3 in the main text). Shown in Supplementary Fig. 8 are the CoV for each duration level (1-64 seconds) for human, Full-frame, "Gaze" and "Shuffled" gaze model duration estimates. The error bars indicate the 95% confidence interval for the CoV, determined from 1000 bootstrapped samples for each duration level and dataset. As can clearly be seen, except at very short durations (< 2 seconds) for both the human and model estimates the CoV is broadly consistent and positioned around a value of 0.5. To confirm this observation, we fit a linear regression to each set (human and three models) of CoVs over duration levels. Shown in Supplementary Fig. 9 is the obtained regression coefficient (slope) and associated 95% confidence interval of the slope estimation for each dataset. Most immediately notable is how small the slope values (indicating change in CoV for additional 1 second in duration) are in each case (Human:-0.0091; Full-frame:-0.0074; "Gaze":0.0004; "Shuffle":-0.0008), consistent with what would be expected given the broadly flat plots seen in Supplementary Fig. 8. Of additional note is that for two out of the three model configurations ("Gaze" and "Shuffle", but not Full-frame), the 95% confidence intervals (CI) of slope include 0, indicating that in these cases there is no evidence to support that the CoV (representing variability in response at each duration level) changes across the tested durations (Full-frame CoV CI:-0.0128 - -0.0020;"Gaze" CoV CI:-0.0032 - 0.0040; "Shuffle" CoV CI:-0.0020 - 0.0004). To appropriately test the equivalent case for the human data, we conducted frequentist and Bayesian repeated measures ANOVAs, with the 13 levels of duration as a within-subjects factor and the CoV as the dependent variable (using JASP, version 0.8.3.1[3]). The frequentist ANOVA revealed no evidence for a difference in CoV across duration levels, $F(7.2, 374.8) = 1.68$, $p = 0.11$, eta-squared $= 0.03$, Greenhouse-Geisser corrected). Supporting this finding, the Bayesian repeated measures ANOVA (using default priors) indicated evidence against a model including duration, $BF_{10} = 0.055$, suggesting that the null model (not including duration as a factor) was around 18 times more likely than the model including duration. These results are consistent with the interpretation reported in the main text on the basis of the standard deviations presented in Fig. 3 and suggest that both human and model estimates in our data are generally consistent with Weber's law/scalar variability.

Effects of regression on different duration ranges Regression to the mean is found in human reports across many perceptual domains and in time perception it is often referred to as Vierordt's law[4]. In recent years there has been much interest in range-limited regression to the mean effects in time perception (e.g.[5,4,6]). Our model relies on applying a regression method (support vector regression) to the accumulated salient perceptual changes so that they can be interpreted in standard units of time (seconds). While we make no claim that the human brain (or any biological system) is doing support vector regression, it is clear that mapping a sensation onto a specific label - time as perceived through accumulated perceptual changes mapped into seconds - is a regression problem. In order to demonstrate how our regression process produces estimates from accumulated salient changes, we trained the regression on a variety of limited duration ranges. Shown in Supplementary Fig. 10, when we train the regression on, for example, only video durations between 4 and 8 seconds, we find regression to the mean for that specific range, with relative over- and under-estimation around the range centre (approximately 6 seconds), and equivalent results for other ranges. These results are, at least superficially, highly similar to those reported in the human behavioural literature (e.g.[5,4,6]) and suggest that regression to the mean effects might be more related to the task of mapping sensation onto appropriate labels, rather than a core feature of time perception as implied in the construction of certain pacemaker models of time[7,2,8].

2 Supplementary Figures

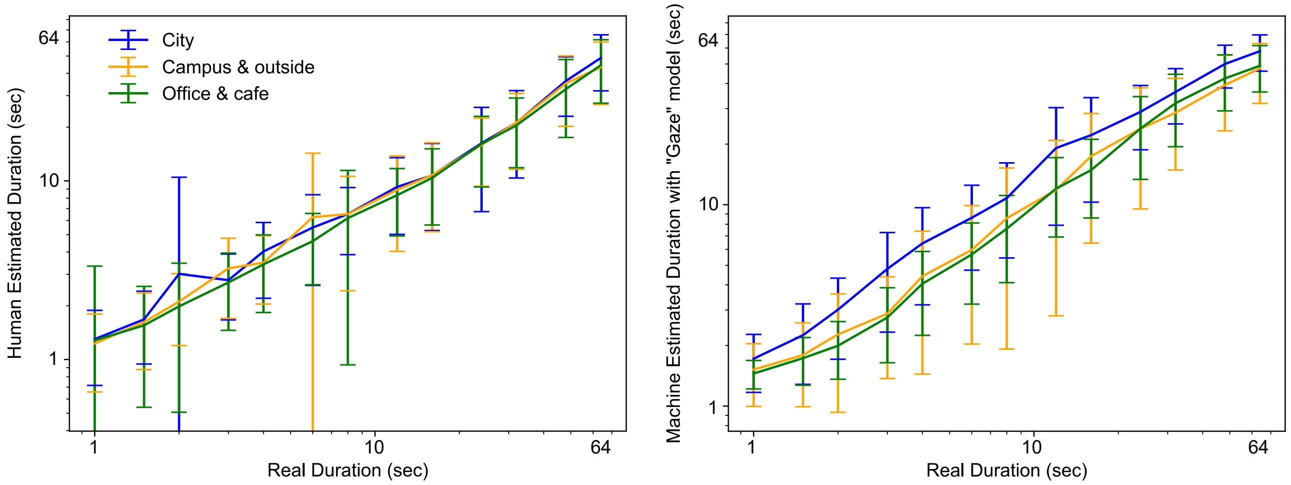

Supplementary Figure 1: Duration estimation for each tested video duration, separated by scene-type, for human (left panel) and model (right panel) experiments. Error bars indicate one standard deviation from the mean. As shown in Fig. 3g and h in the main text, for both humans and the system, city scenes are typically estimated as longer than campus and outside, or office and cafe scenes. The degree of this overestimation is stronger for the system, but the overall pattern of results is the same for human and system estimation.

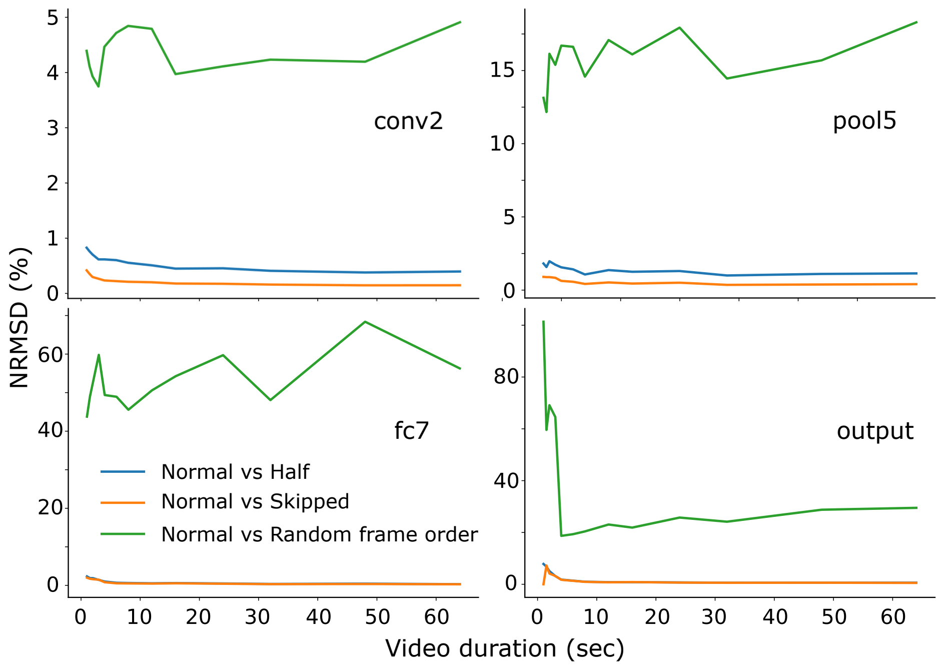

Supplementary Figure 2: Comparison of system accumulation of salient changes depending on input frame rate and composition of the input video. Note that the depicted differences are related to raw accumulated perceptual changes, not duration estimation following support vector regression. Each panel shows the normalised root-mean squared difference (NRMSD) between the accumulated salient changes in the system when given the normal input video at 30 Hz, compared to input videos at half the frame rate, inputs videos with 20% of frames pseudo-randomly skipped, and input videos presented at 30 Hz (same as the normal input videos), but with a random order of frame presentation. The manipulations of frame rate (halving or skipping 20%) had little effect on the accumulated changes (blue and orange lines), while presenting the frames in a random order altered the accumulation of salient changes dramatically (green lines).

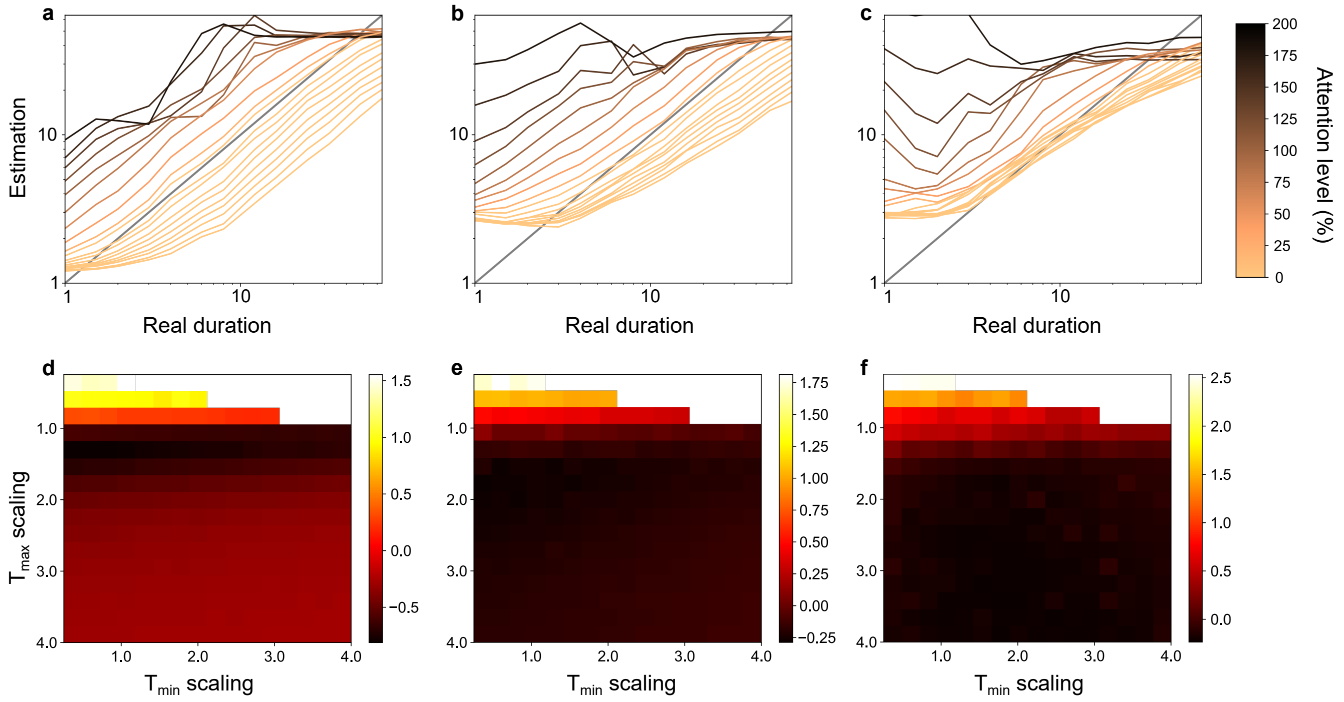

Supplementary Figure 3: Robustness of the temporal attention mechanism. **a** Comparison of system duration estimation at different levels of attention modulation. This level refers to a scaling factor applied to the parameters T_{max} and T_{min}, specified in Table 1 and Equation 1. Each panel shows the performance for a different variant of the model (**ai**:"Gaze", **aii**:"Shuffled" and **aiii**:Full-frame). While changing the attention level did affect duration estimates, often resulting in a bias in estimation (e.g. many levels of the Full-frame exhibit a bias towards over-estimation; darker lines), across a broad range of Attention levels the models (particularly in the "Gaze" model) still differentiate longer from shorter durations, as indicated by the positive slopes with increasing real duration. For the models in Fig. 3 in the main text, the following scalings were used: ("Gaze": 1.20, "Shuffled": 1.10 and Full-frame: 1.06). **b** Normalised root mean squared error (NRMSE) of duration estimations versus real physical durations (**bi**:"Gaze", **bii**:"Shuffled" and **biii**:Full-frame), for different combinations of values for the parameters T_{max} and T_{min} in Equation 1. The gray areas in the heatmap represent combinations of values that cannot be defined. Dotted lines represent the attention threshold scaling used for each model shown in Fig. 3.

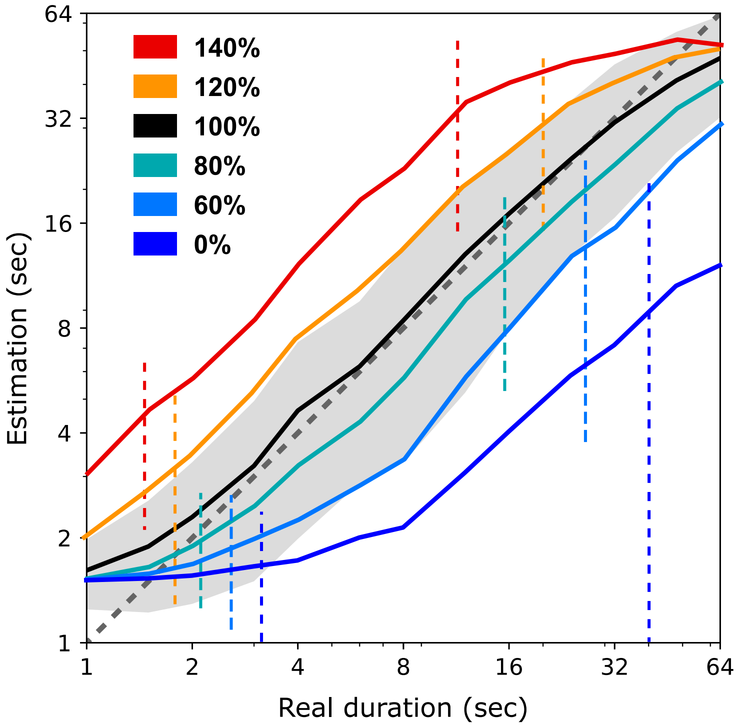

Supplementary Figure 4: Comparison of system estimation with fixed thresholds at different levels of attention modulation. As for estimation with dynamic thresholds (Supplementary Fig. 3), the system can differentiate short from long durations effectively, and modulation of attention level causes a similar pattern of over and underestimation as found with the dynamic threshold. Dotted vertical lines denote one standard deviation from the mean.

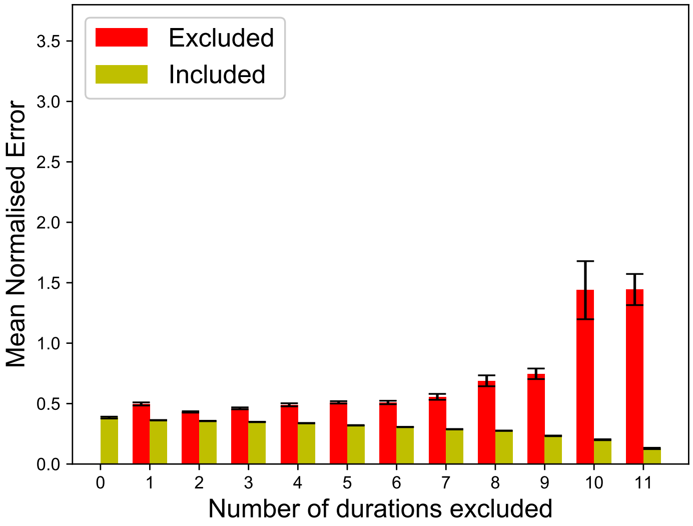

Supplementary Figure 5: Comparison of system performance by means of normalized duration estimation error, when a subset of testing durations were not used in the training process. For each pair of bars, all trials of N randomly chosen durations (out of 13 possible durations) have been excluded (x-axis). The support vector regression was trained on the remaining durations and tested on all durations. The errors for excluded and included trials are reported for each N. Only when excluding a large number of training levels (e.g. 10 out of 13 possible levels) does the estimation error get notably larger. The bars denote the mean performance of 150 training and testing repetitions using all trials for each number of excluded durations, while error bars denote the standard error when estimating the mean from this 150 repetitions.

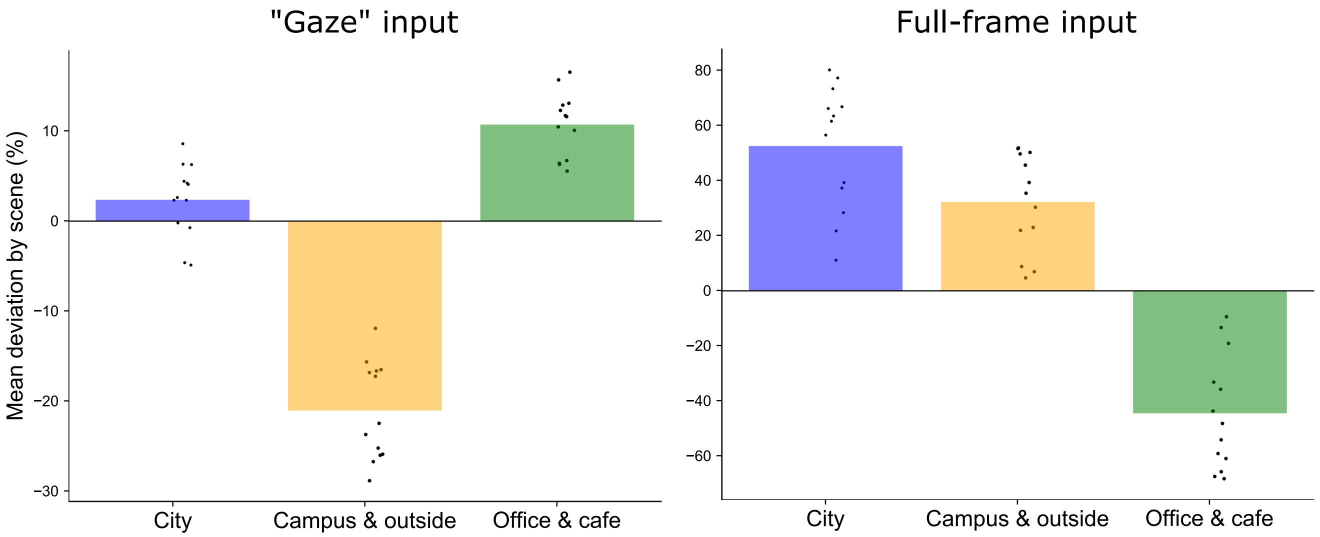

Supplementary Figure 6: Mean deviation of duration estimations relative to mean duration estimation, by scene type. Estimations were produced based on the raw Euclidean distance between video frames, by pixel, rather than using classification network activation. Left panel shows deviation of estimations, based on videos constrained by human gaze ("Gaze" input; as in human and model experiments in the main text), the right panel shows the same based on the Full-frame of the video. Error bars indicate standard error of the mean. For the "Gaze" input: City: 2.35 mean (1035 trials), Campus & outside: -21.03 mean (1167 trials), Office & cafe: -10.70 (2068 trials); Total number of trials 4270. For the "Full-frame" input: City: 52.48 mean (1035 trials), Campus & outside: 32.22 mean (1167 trials), Office & cafe: -44.43 (2068 trials); Total number of trials 4270. For both plots, the baseline attention level was used.

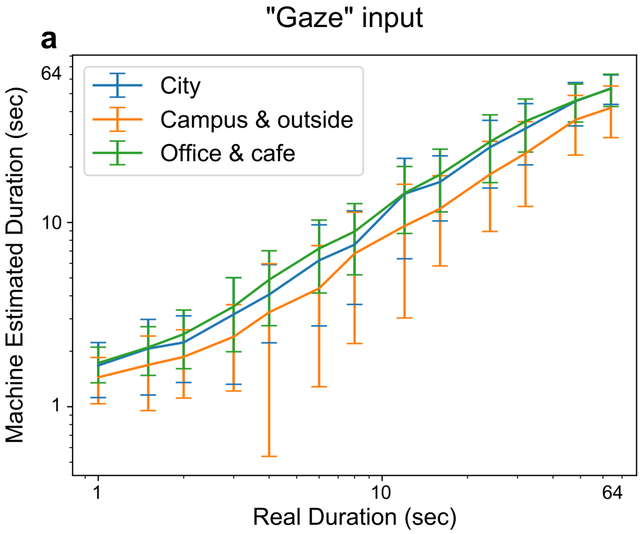

Supplementary Figure 7: Duration estimation for the 13 tested video durations, by scene-type. Estimations were produced based on the raw Euclidean distance between video frames, by pixel, rather than using classification network activation. **a** shows estimations based on videos constrained by human gaze ("Gaze" input; as in human and model experiments in the main text), **b** shows estimations based on the "Full-frame" of the video. Error bars indicate one standard deviation from the mean.

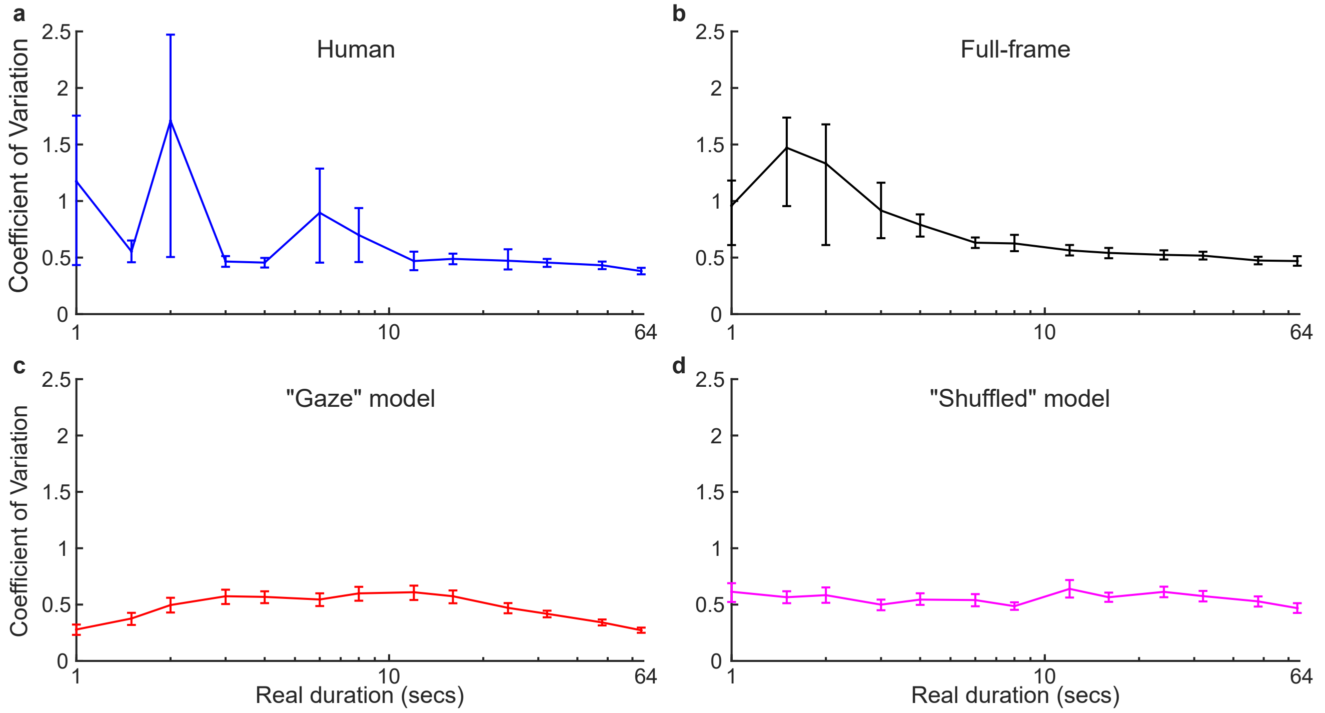

Supplementary Figure 8: Each panel shows the coefficient of variation for each level of duration at which we obtained estimates from Human participants (**a**), using the Full-frame (**b**), “Gaze” constrained (**c**), or “Shuffled” gaze models (**d**). Error bars indicate the bootstrapped 95% confidence intervals. The coefficient is inconsistent at shorter durations (< 2 seconds) for both the Human participants and the model under Full-frame and “ Gaze” configurations, but is broadly consistent across the remaining durations and typically around a value of 0.5 for all sets of estimations. These results are consistent with these dataset exhibiting properties consistent scalar variability/Weber’s law as typically reported for human duration estimations.

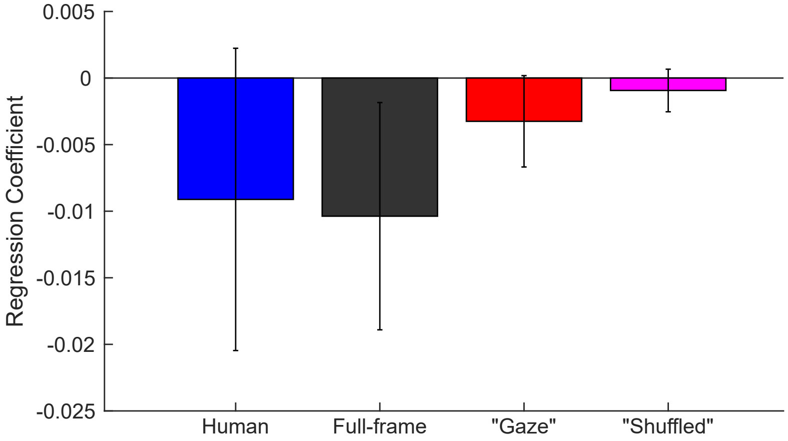

Supplementary Figure 9: Barplot shows the regression coefficients (slope) for linear regressions conducted on the coefficient of variation (CoV) across the 13 tested duration levels for each of our Human estimates, Full-frame, “Gaze”, and “Shuffled” model estimates. The error bars show the associated 95% confidence intervals. See Supplementary Discussion **Estimate variance by duration** for detailed analysis and interpretation.

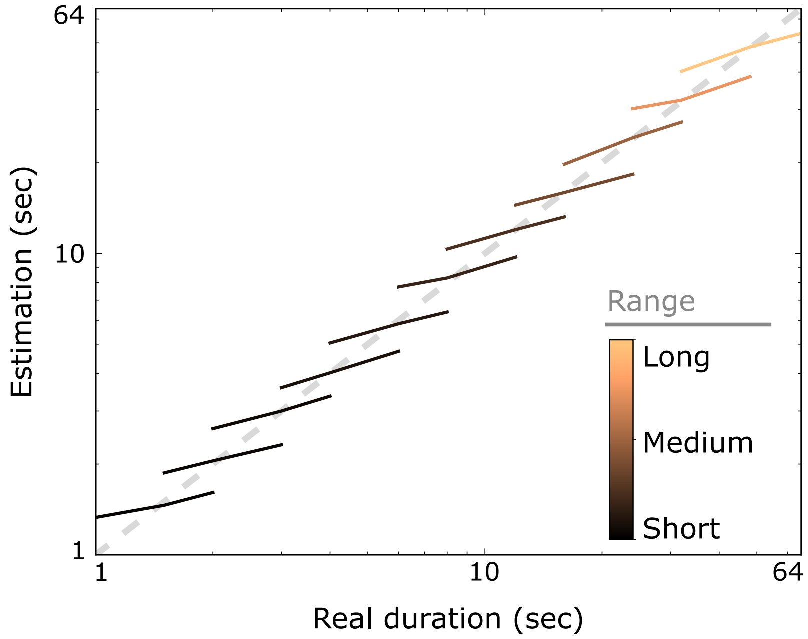

Supplementary Figure 10: Duration estimations when the support vector regression was trained on only a range-limited subset of durations (e.g. 4, 6, and 8 seconds). Each of the lines indicates a single regression training and the non-veridical flattening of the slopes present in each line indicates regression to the mean for that range. Taking a subset of a several partially overlapping ranges together gives a similar pattern of results as those demonstrated in range-limited regression to the mean effects for human reports of time (e.g. Figure 2 in[5])
.

References

[1] Fabian Pedregosa, Gaël Varoquaux, Alexandre Gramfort, Vincent Michel, Bertrand Thirion, Olivier Grisel, Mathieu Blondel, Peter Prettenhofer, Ron Weiss, Vincent Dubourg, et al. Scikit-learn: Machine learning in python. *Journal of Machine Learning Research*, 12:2825–2830, 2011.

[2] Hedderik Van Rijn, Bon-Mi Gu, and Warren H Meck. Dedicated clock/timing-circuit theories of time perception and timed performance. In *Neurobiology of interval timing*, pages 75–99. Springer, 2014.

[3] JASP Team. JASP (Version 0.8.3.1)[Computer software], 2017.

[4] Frederike H. Petzschner, Stefan Glasauer, and Klaas E. Stephan. A bayesian perspective on magnitude estimation. *Trends in Cognitive Sciences*, 19, 2015.

[5] Mehrdad Jazayeri and Michael N Shadlen. Temporal context calibrates interval timing. *Nature Neuroscience*, 13:1020–1026, 2010.

[6] Neil W Roach, Paul V McGraw, David J Whitaker, and James Heron. Generalization of prior information for rapid bayesian time estimation. *Proceedings of the National Academy of Sciences*, 114(2):412–417, 2017.

[7] Matthew S Matell and Warren H Meck. Cortico-striatal circuits and interval timing: coincidence detection of oscillatory processes. *Cognitive brain research*, 21(2):139–170, 2004.

[8] Bon-Mi Gu, Hedderik van Rijn, and Warren H Meck. Oscillatory multiplexing of neural population codes for interval timing and working memory. *Neuroscience & Biobehavioral Reviews*, 48:160–185, 2015.
