# Supplementary material for: Activity in perceptual classification networks as a basis for human subjective time perception
Source: Nat Commun. 2019 Jan 17;10:267. doi: 10.1038/s41467-018-08194-7 (PMC6336826; doi:10.1038/s41467-018-08194-7)
Supplement: Supplementary file 2 — Description of Additional Supplementary Files [file 41467_2018_8194_MOESM2_ESM.docx]

Description of Additional Supplementary Files

**Supplementary Movie 1.** An example video used as stimulus in human and model experiments. This video came from the campus and outside stimulus category.

**Supplementary Movie 2.** An example video used as stimulus in human and model experiments, with direction of human gaze overlaid on the video, indicated by the red box. For models based on human gaze (“Gaze” and “Shuffled”), input to the model on each video frame was constrained by the bounds of this red box. This video also came from the campus and outside stimulus category.
